# Oxidative Stress Parameters as Biomarkers of Cardiovascular Disease towards the Development and Progression

**DOI:** 10.3390/antiox11061175

**Published:** 2022-06-15

**Authors:** Amanda Shen-Yee Kong, Kok Song Lai, Cheng-Wan Hee, Jiun Yan Loh, Swee Hua Erin Lim, Maran Sathiya

**Affiliations:** 1 School of Pharmacy, Monash University Malaysia, Jalan Lagoon Selatan, Bandar Sunway, Subang Jaya 47500, Malaysia; kong.amanda0811@gmail.com; 2 Health Sciences Division, Abu Dhabi Women’s College, Higher Colleges of Technology, Abu Dhabi 41012, United Arab Emirates; lkoksong@hct.ac.ae (K.S.L.); lerin@hct.ac.ae (S.H.E.L.); 3 Faculty of Health and Life Sciences, INTI International University, Persiaran Perdana BBN, Putra Nilai, Nilai 71800, Malaysia; wanhee.cheng@newinti.edu.my; 4 Centre of Research for Advanced Aquaculture (CORAA), UCSI University, Cheras, Kuala Lumpur 56000, Malaysia; lohjy@ucsiuniversity.edu.my

**Keywords:** reactive oxygen species, oxidative stress, oxidant, cardiovascular, biomarkers

## Abstract

Cardiovascular disease (CVD) remains the leading cause of death globally, with unhealthy lifestyles today greatly increasing the risk. Over the decades, scientific investigation has been carried out on reactive oxygen species (ROS) and their resultant oxidative stress based on their changes made on biological targets such as lipids, proteins, and DNA. Since the existing clinical studies with antioxidants failed to provide relevant findings on CVD prediction, the focus has shifted towards recognition of oxidised targets as biomarkers to predict prognosis and response to accurate treatment. The identification of redox markers could help clinicians in providing risk stratification for CVD events beyond the traditional prognostic and diagnostic targets. This review will focus on how oxidant-related parameters can be applied as biomarkers for CVD based on recent clinical evidence.

## 1. Introduction

Cardiovascular disease (CVD) represents an immense burden on health services globally. Every year, CVD contributes to nearly 44% of mortality compared to other non-communicable diseases (WHO, 2021) [1], with the major contributor—ischemic heart disease—giving a prevalence of 126.5 million people [2]. The health statistics reports present that 92.1 million people live with at least one form of CVD in the United States [3]. Comorbidity conditions such as hyperlipidaemia, hypertension, and hyperglycaemia are common in CVD patients which further accelerate the disease progression [4]. There remains growing concern focused on improving the cardiovascular risk stratification and the need for novel biomarkers to provide early diagnosis.

Oxidative stress is assumed to play a pivotal role in cardiac remodelling and is responsible for the promotion and propagation of CVD [5]. Recent research concerning its pathophysiology and development focuses on the breakdown of normal homeostatic systems resulting in oxidative stress. It occurs with the imbalance between the reactive oxygen or nitrogen species (ROS/RNS) and the body’s antioxidant defence systems [6]. The overproduction of ROS may surpass the cellular antioxidant capacity and perturb the equilibrium [7]. These may subsequently induce protein and lipid peroxidation along with DNA mutagenesis that may cause deleterious effects [8]. In addition to eliciting direct cellular damage, oxidative stress also stimulates mitochondrial dysfunction and promotes free radicals’ formation that only exacerbates the disease burden [9].

To date, the precise mechanism by which pro- and anti-oxidative factors influence CVD manifestation and contribute their complications are not fully elucidated [9]. However, in eliciting direct cellular damage, oxidative stress was reported to stimulate mitochondrial dysfunction, dyslipidaemia, and genetic predispositions, and promotes free radicals’ formation that only exacerbates the disease burden [10].

Many clinical and experimental studies provide a comprehensive understanding on antioxidant approaches, such as vitamins C and E to be used concurrently with guideline-recommended drug treatment, but these antioxidants mostly show discouraging results in cardiovascular prognosis [4,11]. There is emerging evidence suggesting that CVDs are associated with or even provoked by oxidised targets. Both animal models and patients with heart failure (HF) constantly showed increased oxidative stress throughout their onset and disease progression [5]. This fact is also well supported by ROS-sensitive pro-hypertrophic and remodelling signalling cascades in the setting of cardiac hypertrophy [8]. Despite the recognition of oxidant biomarkers with their convenience to be used and affordable, available information regarding their prognostic relevance is still limited [10].

**Figure 1:**
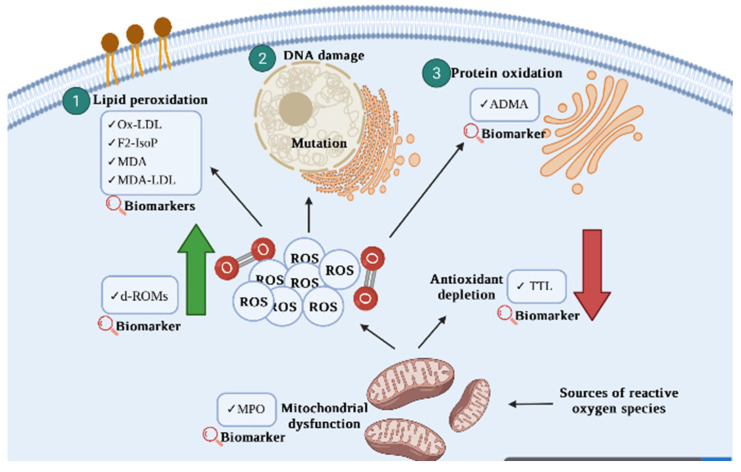
A schematic representation of oxidative stress-related biomarkers for cardiovascular disease that were discussed in the current review. Oxidative stress occurs when there is an imbalance of ROS and antioxidants. The generation of uncontrollable ROS may subsequently induce DNA damage, protein oxidation, and lipid peroxidation, which contribute to the progression and development of CVD.

Nevertheless, the nature of ROS/RNS that are available in the body and specifically target CVD fulfil the essential requirement of a biomarker. The application of oxidant targets with prognostic values may be applied to reflect the disease risk on individuals and potentially be used in precision medicine shortly [4]. In this review, we aim to recapitulate the current knowledge regarding oxidant related parameters so as to estimate their effectiveness as a biomarker for the development and progression of CVD. Figure 1 represents oxidative stress-related biomarkers for cardiovascular disease.

## 2. Oxidant Biomarkers in the Diagnosis and Prognosis of CVD

### 2.1. Myeloperoxidase (MPO)

The primary source of oxidative stress is myeloperoxidase (MPO), a member of the family of heme peroxidases in human vasculature that is primarily secreted by neutrophils and macrophages [12]. Uncontrollable overexpression of MPO is associated with poor cardiovascular outcomes and an increased risk of cardiovascular-related mortality [13]. Mainly, MPO has contributed to the development of plaques in the artery wall with the production of ROS [14]. During the early onset of CVD, neutrophils and macrophages will be activated to synthesise MPO and produce 3-chlorotyrosine (3-Cl-Tyr) and 3-nitrotyrosine (3-NO-Tyr), the by-products that have been widely implicated in the development of atherosclerotic lesions. Both reactive oxidant products could stimulate protease cascades and induce plaque rupture [15]. Over the decades, MPO has been suggested to be used as a powerful diagnostic and prognostic marker for a wide variety of CVD conditions, such as acute myocardial infarction (AMI), coronary artery disease (CAD), and congestive HF [16].

High-density lipoprotein (HDL) is a primary target for oxidative modification in CAD patients [17]. Recent reviews highlighted the ability of MPO in inducing changes and impairing HDL, which resulted in a loss of cardioprotective effect and initiation of pro-inflammatory processes [12,18]. Both 3-Cl-Tyr and 3-NO-Tyr have been shown to impair the ATP-binding cassette transporter (ABCA1)-dependent cholesterol efflux activity which causes excessive cholesterol accumulation in arteries and activates foam cell formation [19,20]. However, a different research finding was demonstrated by Wang and colleagues (2018). The HDL isolated from aortic lesions in CAD patients contains high levels of 3-Cl-Tyr and 3-NO-Tyr. Despite HDL 3-NO-Tyr being identified as the best predictive marker associated with CAD, HDL 3-NO-Tyr did not correlate with 3-Cl-Tyr in MPO plasma, suggesting that the functional 3-NO-Tyr was derived from other RNS instead of MPO action. More studies on MPO-modified HDL are required to explore the association with cardiovascular outcomes before MPO could be used as a target to boost HDL function and provide clinical benefit to patients.

In an attempt to identify AMI in patients presenting angina, Omran and colleagues (2018) have investigated the diagnostic efficiency of plasma MPO levels alone or in combination with creatine kinase (CK)-MB and Troponin I (cTnI) within the first 6 h of disease onset [21]. MPO was found to be the most efficient marker in detecting AMI patients. Interestingly, the combination of MPO, CK-MB, and cTnI was able to provide a sensitivity of 91% and a specificity of 76% in detecting AMI compared to each marker alone. Similar finding was seen in Calmarza and colleagues (2018), whereby the plasma MPO levels significantly increased in ACS patients compared to those with stable angina and yielded its best result at the 6th hour after hospital administration [22].

In the setting of adult male participants with hypertension, Charkiewicz and colleagues (2021) showed high levels of MPO in hypertension men compared to the control groups without the disease [23]. The elevated MPO may induce oxidative stress and trigger endothelial dysfunction which results in increased blood pressure.

Zhang and colleagues (2022) highlighted the association of MPO with inflammatory responses [24]. Several inflammatory markers, such as c-reactive protein, fibrinogen, and neutrophil counts, were significantly increased with the elevation of MPO levels. Further analysis showed that MPO could provide a higher prognostic value in patients > 65 years and NT-proBNP level > 1000 pg/mL, suggesting its role in predicting the occurrence of major adverse cardiovascular events (MACEs) in patients with acute non-ST-segment elevation myocardial infarction (NSTEMI). There was also a positive correlation between MPO and global registry of coronary artery events (GRACE) scores in which high levels of MPO were classified as high-risk groups based on GRACE scores. This subsequently improved the risk classification of NSTEMI and aided in therapeutic approaches.

### 2.2. ox-LDL

Low-density lipoprotein (LDL) is routinely used for cardiovascular risk assessment clinically with high levels indicating greater risk of CVD [25]. In the presence of hydrogen peroxidase, MPO can induce oxidative modification on LDL and release oxidised LDL (ox-LDL) [18]. While the normal LDL only specified one type of receptor, ox-LDL had a higher affinity towards several receptors that greatly aided with its uptake to macrophages and endothelial cells. Together with other underlying metabolic syndromes, ox-LDL further accelerates its deposition in arteries and triggers plaque formation.

Large prospective cohort studies have demonstrated the association of elevated plasma ox-LDL with adverse cardiovascular events [26]. A positive correlation was seen in ox-LDL with the increased severity of CAD [27]. Likewise, Zhao and colleagues (2018) summarised the predictive values of CAD severity by employing six different LDL-related parameters [28]. All of them were raised with increasing severity of CAD, but ox-LDL showed the highest predictive value and was independently associated with CAD severity.

Another experiment performed by Zhao and colleagues (2018) investigated the relationship between ox-LDL and early-onset CAD based on a follow-up study on 1217 untreated patients with angina [29]. Higher levels of ox-LDL were associated with patients with very early CAD (VECAD) compared to the control group. The plasma levels of ox-LDL were independently associated with VECAD, suggesting that ox-LDL could be used as a prognostic predictor for VECAD to aid in disease management.

### 2.3. F2-Isoprostanes (F2-lsoP)

F2-isoprostanes (F2-IsoP) are circulating in bodily fluids, such as serum and urine [30]. Elevated levels of F2-IsoP can be observed in animal models of oxidant injury and human disease defined by elevated ROS. There is increasing evidence focusing on the association of increased F2-IsoP with CVD risk factors, such as hypercholesterolemia, diabetes mellitus, and cigarette smoking, but limited research linking this biomarker to clinical outcomes [27]. Anderson and colleagues (2018) demonstrated an elevation of F2-IsoP metabolite in individuals with incident hypertension [31]. The ability of F2-IsoP in promoting vasoconstriction and inflammation further supports their potential involvement in the development of hypertension.

In a large community-based study conducted by Castro-Diehl and colleagues (2021), urinary F2-IsoP was significantly associated with all causes of mortality, especially in fatal coronary heart disease (CHD) [32]. A similar finding was seen in another large German cohort study performed by Xuan and colleagues (2018) [33]. Urinary F2-IsoP was associated with fatal stroke and older (age ≥ 60 years) obese individuals in all CVD mortality. The ability of F2-IsoP to retain its stability in urine allowed it to assess oxidative stress status in vivo accurately.

### 2.4. Asymmetric Dimethylarginine (ADMA)

Asymmetric dimethylarginine (ADMA) competes with L-arginine to the same binding site of nitric oxide synthase and this affects the oxidative stress process [34]. Cordts and colleagues (2019) suggested that high levels of ADMA in the circulation may reduce nitric oxide production and impaired myocardial relaxation in patients with hypertrophic cardiomyopathy (HCM) [35]. A high concentration of ADMA was also associated with increasing severity of diastolic dysfunction, the early manifestation of HCM. Similarly, Charkiewicz and colleagues supported the association of increased ADMA in hypertension men with endothelial dysfunction [23].

In a meta-analysis conducted by Ye and colleagues (2021), CAD patients had an approximately two-fold increased risk of all causes of mortality and MACEs with the elevation of plasma ADMA [36]. This suggests that ADMA could be a marker to predict deaths and cardiovascular-related outcomes in CAD patients. A similar finding was demonstrated by Xu and colleagues (2019) in which ADMA independently predicted recurrent cardiovascular events in patients with stable CHD after one year of follow-up [37]. Another study by Appel and colleagues (2020) reported that patients with elevated ADMA were at risk of major cardiovascular complications before their non-cardiac operation [34].

### 2.5. Total Thiol (TTL)

Total thiol (TTL) appears early in CVD onset and represents the redox control status of vascular systems [38]. An application of this assay in a pooled case-control study by Xuan and colleagues (2019) showed that increased TTL was associated with incident stroke but only restricted to stroke events that happen in mid-life, ages ranging from 45 to 60 years. Further analysis revealed the association of TTL with fatal MI risk. However, Erdoğan and colleagues (2020) highlighted a different finding in which higher levels of TTL were shown in the control group than in the ascending aortic dilatation (AAD) group [39]. Lower TTL appears to confer a higher risk for AAD development and independently predicts the diameter of ascending aortic.

### 2.6. Derivatives of Reactive Oxygen Metabolites (d-ROMs)

Derivatives of reactive oxidative metabolites (d-ROMs) are a newly discovered marker of ROS and are good in reflecting ROS production of a biological target. Xuan and colleagues (2019) demonstrated an increased level of d-ROMs in the incidence of MI and stroke, but the results were only significant in the male subjects. d-ROMs were also associated with fatal MI risk [38]. Considering a weak correlation between d-ROMs and TTL, researchers proposed the use of a d-ROMs-to-TTL ratio, which provides stronger results compared to the individual markers alone. The d-ROMs-to-TTL ratio was significantly associated with the onset of major cardiovascular events, but this association did not retain when diseases and CRP for the outcome were included.

Nishihara and colleagues (2021) performed an 8-year follow-up cohort study and revealed the usage of d-ROMs in providing prognostic value for risk classification on non-ischemic heart failure (NIHF) [40]. Higher levels of d-ROMs increased the risk of HF-related events.

### 2.7. Malondialdehyde (MDA)

Nitric oxide contributes to oxidative stress by regulating lipid peroxidation and produces MDA as an end-product under physiological conditions [23]. Romuk and colleagues (2019) observed an elevation of MDA with the mortality prediction in chronic HF patients. The risk of mortality and the combined endpoint of death in the research subjects were increased two-fold in the presence of high MDA [41].

Recent novel findings by Boarescu and colleagues (2022) showed the importance of prevalent cardiovascular risk factors with MDA. Hypertension, lipid imbalance, and diabetes mellitus were associated with a slight increase in the concentration of MDA [42]. Similarly, Charkiewicz and colleagues (2021) demonstrated the increase of MDA in male subjects with hypertension. Taken together, these results suggest the role of oxidative stress in vascular endothelial dysfunction and raise the possibility of patients developing cardiovascular complications.

### 2.8. Malondialdehyde-Modified Low-Density Lipoprotein (MDA-LDL)

Malondialdehyde-modified low-density lipoprotein (MDA-LDL) is similar to ox-LDL and represents one of the major products of lipid peroxidation [43]. Research conducted by Amioka and colleagues (2019) revealed the association of high MDA-LDL levels with AMI compared to those with unstable angina. MDA-LDL was able to reflect the presence of vulnerable plaque and independently predict adverse cardiovascular events in ACS patients with successful non-surgical percutaneous coronary intervention (PCI). Not only that, the highest MDA-LDL group was associated with the early onset of ACS. This suggests that the young generation with ACS has poor lipid balance and high oxidative stress. Overall, MDA-LDL could be used as a potent predictor of cardiovascular outcomes and aid in stratifying ACS and AMI patients accordingly. Figure 1 represents oxidative stress-related biomarkers for cardiovascular disease, and studies appraised in this review are summarised in Table 1.

## 3. Molecular Mechanisms of Pro- and Antioxidant towards CVD

Excessive pro-oxidant generation and a reduction in endogenous antioxidant status are associated with cardiovascular diseases, including mitochondrial dysfunction, dyslipidaemia, and genetic predisposition, i.e., autosome recessive hypercholesterolemia [46,47,48].

Intracellular ROS are formed as a by-product of electron transfer and are produced mainly by the mitochondrial respiratory chain. The principal generators of ROS within the respiratory chain are complexes I (NADH: ubiquinone oxidoreductase) and III (ubiquinol: cytochrome c oxidoreductase) [49]. To control the oxidative stress caused by mitochondrial ROS, it uses a complex network of ROS scavenging systems that act together to alleviate the stress. Superoxide dismutases (SODs) convert the highly reactive superoxide radical into hydrogen peroxide, which is then detoxified by GSH-PX and the PRX/Trx systems. Dyslipidaemia is defined as an elevated fasting and postprandial plasmatic concentration of total triglycerides and free fatty acids, with high levels of low-density lipoproteins (LDL) and low levels of high-density lipoproteins (HDL) [50]. Overproduction of reactive oxygen species has been linked to lipid metabolism disorders and is demonstrated to impact the antioxidant state of several organs and lipoprotein levels [51]. Increased superoxide production has been observed in hypercholesterolemic animal models. An increased NADPH oxidase activity is commonly seen as the principal source of superoxide [52,53]. Increased NOX4 transcript levels in hypercholesterolemic animal models are also observed due to transcriptional, posttranscriptional, or epigenetic NOX4 expression regulation [54].

Familial hypercholesterolemia (FH) is an autosomal dominant genetic disorder caused by loss of function, which results in the repression of protein synthesis, causing an extremely high level of low-density lipoprotein cholesterol (LDL-C) leads to atherosclerotic cardiovascular disease [55,56]. In FH patients, oxidative stress is a major factor in the onset and development of atherosclerosis. It is reported that hypercholesterolemia causes the production of superoxide radicals, which inhibits the activity of endothelial nitric oxide synthase (eNOS), lowering NO bioavailability as an antioxidant resulting in an inflammatory response in the artery wall [57,58].

## 4. Clinical Significance of Oxidative Stress Parameters Associated with CVD

Biomarker development for prognostic or predictive of CVD is critical for the evolution of treatment options [59]. Several effective research attempts have been conducted to determine the efficacy of various oxidative stress biomarkers. Antioxidants such as thiol are being investigated as a tool for avoiding oxidative stress in cells. It is reported that thiols transform into reversible disulphide structures during oxidative stress and are then reduced back to thiol groups when the oxidative stress is removed. A study by Stoppa-Vaucher and colleagues (2012) investigating teenagers with essential hypertension reported higher disulphide levels indicating increased oxidative stress [60]. In addition, another study comparing the body mass index of matched controls showed that children with hypertension had a worse antioxidative capability due to considerable glutathione depletion [61]. Adipokines such as the Interleukins are also reported as an oxidative biomarker for determining CVD risk [62,63,64]. Type 1 diabetes mellitus is reported as the major risk factor for accelerated atherosclerosis and vascular problems [65]. This data highlights the importance of proper inflammatory monitoring towards efficient interventions. Novel biomarkers, such as urine-tocopherol, a vitamin E metabolite, are being researched as they are considerably higher in children with type 1 diabetes [66].

## 5. Insights into ROS Detection Technology

The detection of reactive oxygen species (ROS) has attracted interest in academic, medical, and industrial settings, particularly in elucidating its pathological mechanism and diagnosis [67]. To date, there are no universal methods for detecting ROS directly and consistently; the electron spin resonance (ESR) [68,69], mass spectrometry (MS) [70], spectrophotometry [71,72], high-performance liquid chromatography (HPLC) [73], fluorescence spectroscopy [74], and electrochemical techniques have been elucidated as an effective technique. Recent advancements in screening technologies enabled the development and enhancements of these technologies. A recent study by Jiang and colleagues (2022) developed a high-throughput fluorescent sensors to detect H_2_O_2_ in live cancer cells [75]. Another recent study by Miripour and colleagues (2022) detected the presence of ROS/H_2_O_2_ in the peripheral blood of cancer patients using a real-time electrochemical test [76]. The researchers successfully identified ROS/H_2_O_2_ levels, indicating that these techniques could possibly use for detecting of ROS in CVD.

## 6. Challenges and Limitations in Targeting Oxidative Stress and CVD Biomarker

Oxidative stress contributed to the progression of various CVD, but controversy flared up with the application of antioxidant therapies in human clinical trials [77]. The effectiveness of antioxidants is generally influenced by the involvement of specific oxidants in disease pathology [78]. Mostly, oxidative stress plays a secondary role in disease causation, and, hence, targeting oxidative stress may not be entirely beneficial to disease progression. The short lifespan of ROS remains the main challenge and makes them difficult to measure accurately [79]. While approaches using the molecule that emits fluorescence such as dihydroethidium (DHE) and MitoSOX have been developed to detect ROS, their sensitivity and specificity results were discouraging since these molecules also appear in other non-specific redox reactions [46]. More novel approaches or validation studies with large human clinical trials focusing on current existing oxidant biomarkers are highly anticipated in breaking through the seemingly dead-end situation.

## 7. Conclusions

ROS represents a pivotal function in the cardiovascular system, but overproduction of ROS may exceed the capacity of the antioxidant defence systems, causing oxidative stress and inducing adverse cardiovascular outcomes. Despite the emerging attention on oxidant biomarkers as crucial adjuncts in diagnostic and prognostic usage, there is still a long way for their validation in clinical application. More work is required in large clinical trials to demonstrate the value of oxidant markers in addition to the established models of cardiovascular risk prediction. This study presenting oxidant biomarkers potential may guide future efforts for early cardiovascular diagnosis and provide clinical benefit for patients. Future research should focus on the identification of improved biomarkers that integrate the functionality of these biomarkers into a standardised clinical chemistry test.

## Figures and Tables

**Table 1:** Summary of studies performed on oxidative stress biomarkers with cardiovascular disease.

Oxidant Biomarkers	Type of Disease	Type and Number of Samples	Type of Study	Findings	References
MPO	Coronary artery disease	80 Plasma samples	Cross-sectional case-control	Isolated HDL contains high levels of 3-Cl-Tyr and 3-NO-Tyr No correlation between Cl-Tyr and 3-NO-Tyr in MPO plasma	[17]
	Acute myocardial infarction	120 Serum samples	Cross-sectional	MPO was the most efficient marker in detecting AMI Combination of MPO, CK-MB, and cTnI gave 91% sensitivity and 76% specificity for AMI within the first 6 h of disease onset	[21]
	Acute coronary syndrome	83 Plasma samples	Transversal analytical (cross-sectional)	Plasma MPO level increased in ACS patients MPO concentration yield its best result at the 6th hour	[22]
	Arterial hypertension	53 Serum samples	Cross-sectional	High level of MPO was associated with hypertension male subjects	[23]
	Non-ST segment elevation myocardial infarction	271 Plasma samples	Prospective single-center cohort	MPO associated with inflammatory responses Higher prognostic value with MPO in patients > 65 years and NT-proBNP level > 1000 pg/mL Positive correlation between MPO levels and GRACE score	[24]
	Hypertrophic cardiomyopathy	Human cardiomyocytes	-	MPO inhibition alleviates the relaxation defect in hypertrophic iPSC-CMs through MYBPC3 phosphorylation	[44]
ox-LDL	Coronary artery disease	1977 Fasting venous blood samples	Single center observational	ox-LDL showed the highest predictive value for increasing severity of CAD among the other five LDL-related parameters ox-LDL was independently associated with CAD severity	[28]
	Very early coronary artery disease	1217 Plasma samples	Observational Cohort	ox-LDL elevated in patients with VECAD compared to the controls ox-LDL was independently associated with VECAD	[28]
	atherosclerosis	high-fat diet-fed atherosclerosis-prone apolipoprotein E-deficient mice	In-vitro study	ox-LDL could induce endothelial injuries by inhibiting cell proliferation and promoting apoptosis	[32]
F2-lsoP	atherosclerosis	Framingham Offspring Study participants	prospective community-based study	Reflect on aging process	[45]
	Incident hypertension	897 Women urine samples	Cross-sectional case-control	F2-lsoP metabolites increased in individuals with incident hypertension	[31]
	Fatal coronary heart disease	2314 Urine samples	Prospective community-based	Urinary F2-lsoP associated with all causes of mortality, especially in fatal CHD	[32]
	Fatal Stroke	9949 Spot urine samples	Population-based cohort	Urinary F2-lsoP associated with fatal stroke Urinary F2-lsoP significantly associated with older obese individuals in all cardiovascular endpoints F2-lsoP retained its high stability in urine	[33]
ADMA	Hypertrophic cardiomyopathy	215 Plasma samples	Retrospective cross-sectional	High level of ADMA was associated with increasing severity of diastolic dysfunction in patients with HCM	[35]
	Arterial hypertension	53 Serum samples	Cross-sectional	High level of ADMA was observed in men with hypertension	[23]
	Coronary artery disease	-	Meta-analysis	Elevation of ADMA doubled the risk of all causes of mortality and MACEs in patients with CAD	[36]
	Recurrent cardiovascular events (1-year follow-up)	(36 cases, 36 controls) Serum samples	Prospective nested case control	ADMA independently predicted recurrent cardiovascular events in patients with stable CHD	[37]
	Preoperative cardiovascular complications	269 Non fasting plasma samples	Single-centre, prospective, randomised, double-blind	Elevated ADMA increased the risk of major cardiovascular complications in preoperative period	[34]
TTL	Myocardial infarction and stroke	(476 MI and 2380 control, 454 stroke and 2270 control) Serum samples	Pooled case-control	Elevated TTL was associated with incident stroke but only restricted to stroke events that happen in mid-life TTL was associated with fatal MI risk	[38]
	Ascending aortic dilatation	184 Plasma samples	Cross-sectional	Higher levels of TTL were shown in control group Lower TTL conferred higher risk for AAD development TTL independently predicted the diameter of ascending aortic	[39]
d-ROMs	Myocardial infarction and stroke	(476 MI and 2380 control, 454 stroke and 2270 control) Serum samples	Pooled case-control	Increased level of d-ROMs was associated with MI and stroke incidence, but only significant in the male subjects d-ROMs was associated with fatal MI risk d-ROMs-to-TTL ratio was proposed and significantly associated with the onset of major cardiovascular events	[38]
	Non-ischemic heart failure	201 Serum samples	Single-centre, retrospective	d-ROMs could provide prognostic value for NIHF risk stratification High level of d-ROMs was associated with increased risk of HF-related events	[40]
MDA	Chronic heart failure	774 Serum samples	Prospective cohort	Increased levels of MDA could predict the mortality in chronic HF patients MDA was associated with double the risk of mortality and combined endpoint of death	[41]
	Cardiovascular risk factors (Hypertension, lipid imbalance & diabetes mellitus)	28 Wistar-Bratislava white male rats	Experimental animal	Hypertension, lipid imbalance and diabetes mellitus were associated with a slight increase in MDA	[42]
	Arterial hypertension	53 Serum samples	Cross-sectional	Elevated MDA was associated with male subjects with hypertension	[23]
MDA-LDL	Acute coronary syndrome, Acute myocardial infarction	370 Serum samples	Retrospective, single-centre study	High levels of MDA-LDL were associated with AMI compared to those with unstable angina MDA-LDL independently predicted adverse cardiovascular events in ACS patients after undergoing PCI The highest MDA-LDL group was associated with early onset of ACS	[43]
